# 2D-SWE of the Metacarpophalangeal Joint Capsule in Horses

**DOI:** 10.3390/vetsci9090478

**Published:** 2022-09-04

**Authors:** Giulia Guerri, Adriana Palozzo, Paola Straticò, Vincenzo Varasano, Gianluca Celani, Paola Di Francesco, Massimo Vignoli, Lucio Petrizzi

**Affiliations:** 1 Veterinary Teaching Hospital, Faculty of Veterinary Medicine, University of Teramo, Località Piano D’Accio, 64100 Teramo, Italy; 2 Arma dei Carabinieri, Viale Romania, 45, 00197 Roma, Italy

**Keywords:** shear wave elastography, elastosonography, osteoarthritis, fetlock, horse

## Abstract

**Simple Summary:**

Osteoarthritis of fore fetlock in horses is a common disorder that causes owners to complain. It leads to changes in periarticular soft tissue composition. Diagnosis is achieved with radiography and ultrasonography when the disorder is already established. Two-dimensional shear wave elastography (2D-SWE) is an ultrasound-based technique that provides information about tissue composition and elasticity, measuring the velocity of shear waves and tissue stiffness. This study aimed to evaluate the feasibility of 2D-SWE of the fore fetlock joint capsule in horses free and affected from osteoarthritis and to compare their elastographic patterns. The technique was reproducible and repeatable. Age and capsule thickness did not seem to influence the elastographic variables in any group. Longitudinal scan, usually preferred by scientists because it produces fewer artifacts, did not provide significant results. Significant differences were found only in transverse scans, with horses with osteoarthritis having less stiff joint capsule. Poor sensitivity and reproducibility were found. Currently, 2D-SWE of the fetlock joint capsule is not suitable for clinical application. The promising results of other studies suggest that future research should be performed to establish a correlation with MRI or synovial fluid markers which are considered gold standard for diagnosis of osteoarthritis.

**Abstract:**

(1) Two-dimensional shear wave elastography (2D-SWE) employs an ultrasound impulse to produce transversely oriented shear waves, which travel through the surrounding tissue according to the stiffness of the tissue itself. The study aimed to assess the reliability of 2D-SWE for evaluating the elastosonographic appearance of the distal attachment of the fetlock joint capsule (DJC) in sound horses and in horses with osteoarthritis (OA) (2). According to a thorough evaluation of metacarpophalangeal joint (MCPJ), adult horses were divided in a sound Group (H) and in OA Group (P). Thereafter, a 2D-SWE of MCPJs was performed. Shear wave velocity (m/sec) and Young’s modulus (kPa) were calculated independently by two operators at each selected ROI. Statistical analysis was performed with R software. (3) Results: 2D-SWE had good–excellent inter-CC and intra-CC in both groups. Differences in m/s and kPa between Groups H and P were found in transverse scans with lower values in Group P. No correlation with age or DJC thickness was found. (4) Conclusions: 2D-SWE was repeatable and reproducible. In Group H, DJC was statistically stiffer than in Group P only in transverse scan. The technique showed poor sensitivity and specificity in differentiating fetlocks affected by OA.

## 1. Introduction

Osteoarthritis (OA) is a common orthopedic disorder that causes early retirement from competition of sport horses [1]. Diagnosis of OA is classically achieved through radiographic and ultrasonographic examination, although nowadays MRI is considered the most reliable noninvasive diagnostic tool [2]. Nevertheless, none of these methods provides information about viscoelastic properties of tissues [3]. Capsulitis and synovitis have been recognized as initial triggers of OA, and they lead to changes in tissue characteristics, modifying the type of collagen and composition of the capsule itself and its surrounding soft tissue [4,5]. Sonoelastography is based on a physical compression of tissue that produces its displacement according to its stiffness [6]. Due to the difference in elastic properties, it can allow differentiation between normal and diseased tissues [7,8]. Differently from strain elastography, two-dimensional shear wave elastography (2D-SWE) produces automatic generation of shear waves and analysis of their velocity within tissue according to its viscoelastic properties [9,10]. It produces impulses, evaluates their propagation within the examined tissue, and finally generates an elastogram, which is a colored representation of tissue response to the generated impulse [11]. So far 2D-SWE have been used to investigate breast and thyroid lesions and liver fibrosis [12,13,14,15]. While strain elastography (SE) gained popularity as an aid to investigate tendon and ligaments [16,17,18,19,20,21,22] both in human and veterinary orthopedics, 2D-SWE musculo-skeletal application is still limited, particularly in veterinary medicine [23,24,25,26]. Little is known about joint elastography, especially about the synovium and its aspect in normal patients versus those with inflammatory arthropathy. Two variables must be considered for 2D-SWE: shear wave speed, expressed as meters per second (m/s), that measures the propagation of the particles that oscillated transversely to the wave propagation, and tissue stiffness, which is estimated by the Young’s modulus (*E*) (kPa) and measures the resistance of a material to an external unidirectional compression. These two unit of measures are linked together by the relationship *E* = 3*ρc_s_*^2^, where *ρ* is tissue density and *c_s_* represents shear wave speed [27,28]. They are linearly correlated only in case of tissue density equal to 1, which is not the case in the alive tissue [29]. This study aimed to assess feasibility of 2D-SWE on the distal attachment of fore metacarpophalangeal joint capsule (DJC) in horses, and to compare its elastosonographic characteristics among sound horses and horses with OA of the joint. Our hypothesis was that OA and capsule fibrosis would lead to a different elastographic appearance. 

## 2. Materials and Methods

All the study procedures were approved by the local Ethical Committee (Prot. N. 11/2019). The horses were prospectively recruited among those presented at the Veterinary Teaching Hospital of the University of Teramo, after an orthopedic examination by a board-certified equine surgeon (LP). Horses with flexural deformities were excluded from the study. Those that were free of lameness and of any sign of OA of both metacarpophalangeal joints after a radiographic and ultrasonographic assessment were allocated to Group H. Lame horses, after a negative digital palmar nerve block and positive low palmar nerve block and/or intra-articular anaesthesia of the metacarpophalangeal joint, were allocated to Group P [30]. 

For diagnostic purposes, intravenous sedation was provided to all horses (xylazine 0.5 mg/kg) (Nerfasin, ATI). Assessment of fore metacarpophalangeal joints for abnormal findings of bony or soft tissue structures was achieved with a radiographic and ultrasonographic examination [31]. A score was assigned to each joint: the radiographic one was based on the evaluation of osteophytosis development in a four-degree system for judgement (0-3), where 3 corresponded to the worse stage of OA [32]; the ultrasonographic one ranged from 0 to 14 and was assigned to each joint according to the presence of osteochondral irregularities, increased and heterogeneous plica, increased thickness of the DJC [33] (Supplementary material Table S1).

Radiographic evaluation of both fore fetlocks was performed with a M.T. Medical Technology CS01MS equipment in the standard views (latero-medial and dorso-palmar). A high frequency linear probe (8.5–10 MHz) connected to an ultrasound system (Logiq S8XD Clear, GE) was used for the ultrasonographic assessment of the same joints in the longitudinal and transverse views. 

2D-SWE of the DJC at the dorsal aspect of the proximal phalanx (P1) was performed by two experienced operators in transverse and longitudinal scans with the limb in a weight-bearing position. The probe was held still, and 5–10 cycles were recorded for each scan by the software. Elastosonographic images were independently and randomly analyzed by two observers who were blinded to the group to which the horses were assigned. The region of interest (ROI) (10 mm diameter) was placed over the DJC. The elastosonographic software calculated the velocity (m/s) and the Young’s modulus (kPa) of the DJC for each scan.

Every measurement was repeated 3 times by two operators and intraoperator agreement was evaluated. Normality of data was assessed with the Shapiro-Wilk test. Non-parametric statistics was used to compare data collected by the two operators or by the same operator (the Mann–Whitney U test and the Friedman test), or between left to right limbs (Wilcoxon test). The interclass correlation coefficient (inter-CC) and intraclass correlation coefficient (intra-CC) were also calculated for m/s and kPa in both groups. Intra-CC estimated were calculated based on a mean-rating (k = 3), absolute agreement, 2 way random-effect model while the Inter-CC were calculated based on a mean-rating (k = 2), consistency, 2 way random-effect model. A receiver operating characteristic (ROC) curve and the area under the curve (AUC) were calculated. The optimal cut-off value for the velocity and Young’s modulus was selected at the point with highest sensitivity and specificity.

Correlation between the variables m/s-kPa and DJC thickness was analyzed with a Pearson’s correlation test. 

Data were collected on digital worksheets (Excel, Microsoft) and analyzed with open statistical software [34]. Statistical significance was set for *p* < 0.05.

## 3. Results

**Figure 1:**
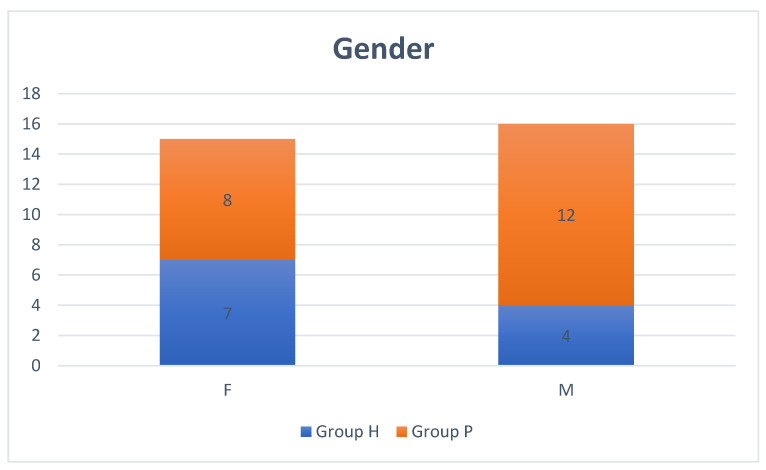
Gender distribution in Group H (blue) and P (orange) (F: female patients; M: male patients).

Thirty-one horses were included in the study, mixed in age and breed. Eleven were assigned to Group H and 20 to Group P. In Group H 8 horses were Standardbred, 1 Spanish, 1 Frisian, 1 mixed breed. In Group P 8 horses were Standardbred, 7 American Saddle, 4 Thoroughbreds, 1 Haflinger. Median age was 10 (range 2–19 years) and 10 (range 2–20 years), respectively in Group H and Group P (*p* = 0.81). The gender distribution in the two groups is summarized in Figure 1. 

In Group P, 11/20 (55%) horses had bilateral OA, 4/20 (20%) had right limb and 5/20 (25%) left limb involvement. 

The most represented radiographic score was 1 (56% of cases in the left limb and 73% on the right limb), followed by grade 2 (25% in the left limb and 27% in the right limb). Grade 3 was shown only in 19% of cases, always in the left limb. The mean total scores for the ultrasonographic examination were 3.96 ± 1.95 on the left forelimb and 3.03 ± 1.55 on the right forelimb. Osteochondral irregularities were reported in twenty-one (68%) limbs (mean score 0.85 ± 0.85). Increased and heterogeneous plica was observed in twenty-six limbs (84%) (mean scores 0.57 ± 0.66 and 0.71 ± 0.66). The joint capsule thickness was appreciated in twenty-three cases (74%) and hypoechogenicity in twenty-nine (94%) (0.66 ± 0.61 and 0.87 ± 0.50, respectively). In twenty-seven cases (87%), the joint capsule insertion was moderately irregular (mean score of 0.87 ± 0.50).

Group H showed lower values for mean thickness of DJC compared to Group P (Table 1).

In both groups the intra-CC was excellent (>0.75) [35]. The inter-CC was always excellent in Group H, while in Group P it was good (0.57) in the evaluation of velocity in transverse scan and excellent for the other variables (m/s in longitudinal scan and kPa in both scans). 

**Figure 2:**
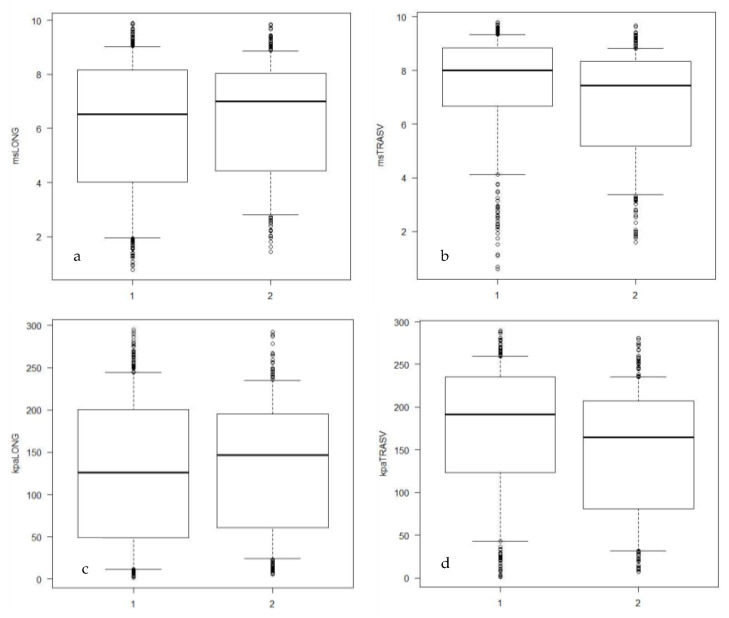
(**a**–**d**): Box-and-whiskers plots showing the distribution of the variables velocity (m/s) and Young’s modulus (kPa) in longitudinal and transverse scan. msLONG: velocity in logitudinal scan; kPaLONG: Young’s modulus in longitudinal scan; msTRASV: velocity in transverse scan; kPaTRASV: Young’s modulus in transverse scan; 1: Group H; 2: Group P.

Significant differences were detected comparing m/s and kPa in transverse scans (*p* < 0.05; Mann–Whitney U test) between Group H and Group P (Table 2), with higher values of both variables in Group H compared to Group P (Figure 2a–d). Longitudinal scans data did not show any significative result (*p* > 0.05; Mann–Whitney U test), although lower median values were calculated for Group H.

**Figure 3:**
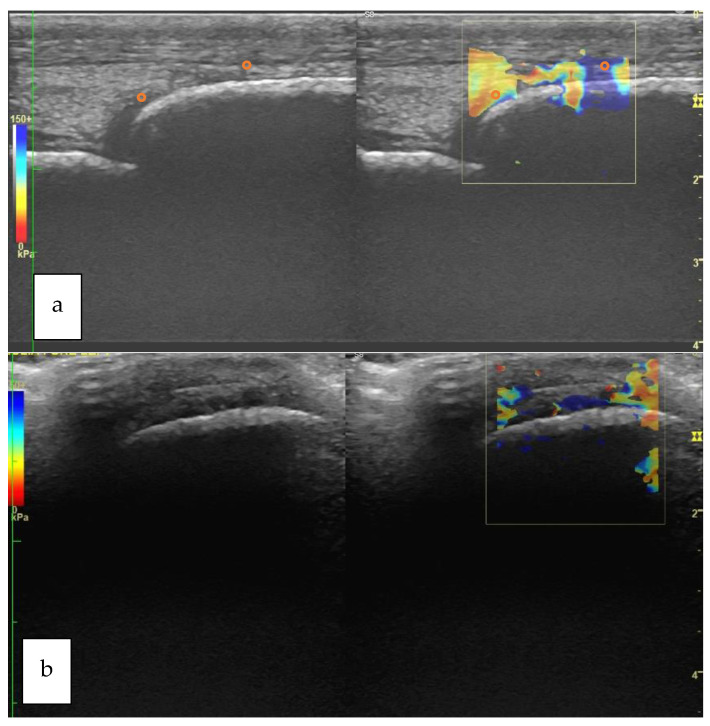
Longitudinal (**a**) and transverse (**b**) view of the metacarpophalangeal joint of a horse belonging to Group H. The orange circle that is superimposed to the elastogram shows the positioning of the ROI. The B-mode corresponding to each elastogram is shown on the left of each box (Group H: horses not affected by osteoarthritis; ROI: Region of Interest).

**Figure 4:**
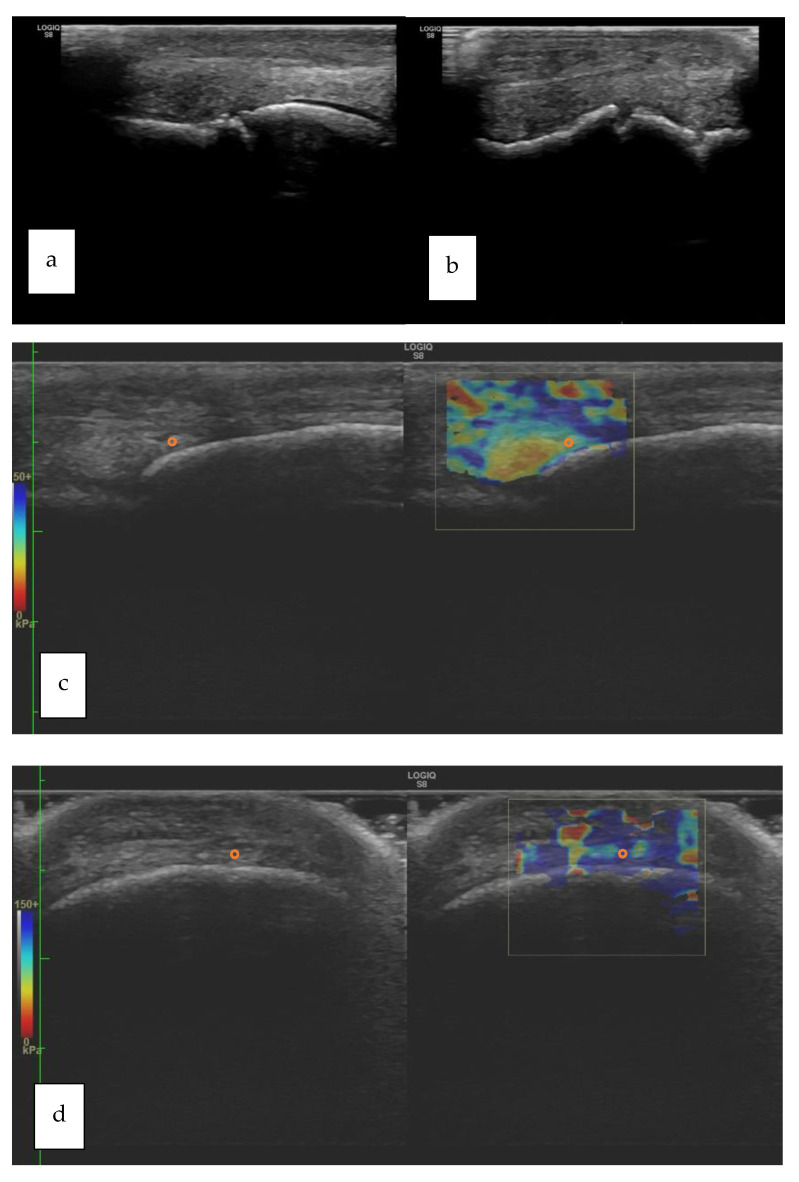
(**a**–**d**): longitudinal (**a**) and transverse (**b**) B-mode images of a right fore fetlock joint showing bone remodeling of the third metacarpal bone (McIII); longitudinal (**c**) and transverse (**d**) views of the metacarpophalangeal joint of a horse belonging to Group P. The orange circle that is superimposed to the elastogram shows the positioning of the ROI. The B-mode corresponding to each elastogram is shown on the left of each box (Group P: horses affected by osteoarthritis; ROI: Region of Interest).

Typical longitudinal and transverse elastograms are shown in Figure 3 for Group H and in Figure 4 for Group P. 

No differences in left and right limb values could be appreciated nor in Group H or Group P (*p* > 0.05; Wilcoxon test) (Table 3).

In the case of monolateral OA in Group P, when analyzing velocity (m/s) and Young’s modulus (kPa) between affected and unaffected limbs in Group P, statistically significant differences were found only in longitudinal scans (*p* < 0.05; Wilcoxon test) with higher value of both variables in the affected limb (Table 4).

The AUC for each variable, the 95% IC, and the optimal cut off value are shown in Table 5. The values indicate that the test has poor performance (AUC 0.6–0.7) in transverse scan and very poor performance (AUC < 0.6) in longitudinal scan.

No correlation was found between DJC thickness and velocity or Young’s modulus, or between age and velocity or Young’s modulus. 

## 4. Discussion

This study aimed to assess feasibility of 2D-SWE on the distal attachment of fore fetlock joint capsule (DJC) in horses, and to compare the elastosonographic characteristics of horses affected and not affected by OA of the joint. In other conditions, mainly soft tissues, such as liver and breast disorders, the 2D-SWE is considered a promising technique [27,28,36], and in clinical conditions tissue elasticity imaging may add useful information to conventional ultrasound B mode examination [27]. Our hypothesis was that 2D-SWE was easy to apply over the dorsal fetlock region to evaluate capsule stiffness in sport horses, with a good degree of reproducibility and repeatability. Moreover, we hypothesized that it could discriminate between fetlock affected and not affected by OA. Our results showed that this technique was reliable in term of repeatability and reproducibility when two expert blind operators were involved, but not reliable to the second aim. Indeed, the discrimination of sound and non-sound joints was possible only in transverse scans, which were less reliable for tissue anisotropy, and the degree of sensitivity and specificity of the techniques was poor. 

2D-SWE of the DJC of fore fetlocks in horses showed excellent repeatability in both groups, while reproducibility in Group P was good in transverse and excellent in longitudinal scan. Similar results were obtained in a study that evaluated strain elastography of the DJC, probably due to a large variability of the elastosonographic aspect of the region in horses affected by OA [16], and in a study that analyzed spontaneous lesions of equine SDFT (Superficial Digital Flexor Tendon) with SE [37]. A good quality B-mode image is indeed essential to correctly place the ROI over the elastogram, avoiding the inclusion of undesired structures, and good repeatability of measurements is strictly associated to it. 

When we compared the velocity and the Young’s modulus between the groups, significative differences were appreciated only in transverse scans, with higher values in group H. Since transverse scans are usually considered less reliable than longitudinal [17,19,24,35], this result must be cautiously interpreted. We did not use any standoff pad to reduce artifacts occurrence (i.e., reverberation artifacts) [21] that were more frequent using the pad itself. The absence of a pad could have been responsible of a higher pressure over the region to maximize probe contact. Tissue compression causes increased 2D-SWE measures, with a duplication of the values with a 10% higher pressure [38]. Since we did not measure the pressure that was applied or did not use a mounted transducer [39], we are not able to quantify the effect of this variable on our results. Moreover, since joint capsule can be considered transversely isotropic due to its fiber alignment, as in muscles and tendons [29,40], only a longitudinal orientation of the probe is able to produce results that are consistent with the mechanical properties of the tissue. 

In accordance to previous literature, since velocity is a function of the angle between the transducer and the main axis of the fibers [41], longitudinal scans provided faster transmission of the impulse due to tissue and ultrasound waves orientation [40] and better image quality. Nevertheless, longitudinal orientation was not able to discriminate affected joints between the groups. This result is in contrast with what is reported in literature where elastographic longitudinal views are usually preferred to transverse for the fewer artifacts and more homogeneous results [16,17,19,24,35]. 

Although 2D-SWE already finds many clinical application in human medicine as an adjunctive diagnostic tool in breast [12], and liver [14,42,43,44] disorders, results and cut-off value used to differentiate between normal and pathologic conditions are still under debate. In orthopedics the Achille’s tendon characteristics have been widely investigated with disomogeneous results [6,45]. In our study, 2D-SWE demonstrated low sensitivity and specificity with AUC ranging from 0.5 to 0.6 in both scans. The cut off values calculated to discriminate between horses with OA and those without OA combining the optimal sensitivity and specificity have for this reason a limited value. The large number of patients with grade 1 OA assigned to Group P may have affected the sensitivity and specificity of the technique. Nevertheless, we did not have enough patients with a higher grade of OA to analyze their SWE trend. Because of the cross-sectional observational nature of the study, no follow-up of patients is available. For this reason, we may have lost data related to the modification of the clinical condition or of the elastographic appearance of the region during time in the recruited patients. As Dirrichs and colleagues demonstrated, 2D-SWE was able to monitor the healing process of naturally occurring tendinopathies in human patients in a longitudinal double-blinded study [46]. The possibility to follow the healthy patients in a longitudinal study should determine whether 2D-SWE is able to differentiate early subclinical conditions, whereas worsening of the clinical condition in patients affected by OA could be related to changes of the elastographic pattern of the joint capsule.

Significant differences were found in Group P when the not affected joint was compared to the affected joint, with lower values of velocity and the Young’s modulus in the first compared to the latter in longitudinal scan. A different elastographic aspect of the unaffected compared to the affected joint in horses belonging to group P may be due to a different pathologic involvement probably related to the increased load of the unaffected joint. 

Although the DJC thickness was statistically lower in Group H, when 2D-SWE measurements were correlated with DJC thickness, the technique did not detect any correlation in any group.

Age has been widely investigated in human medicine for its potential influence on tendons and ligaments stiffness with different results [47,48,49,50]. In our study 2D-SWE measurements were not correlated with age in any of the two groups. As previously said, a longitudinal assessment of patients over time would highlight if there were an age-related change of the elastographic aspect of the DJC of fore fetlock in horses.

The controversial results within literature regarding elastosonography may be related to the strong dependency of the technique on ROI positioning and dimension, on the preset used for the examination, on the pressure needed to perform the exam, on tissue orientation and degree of tension within the tissue itself (contraction vs extension) [51]. The influence of these parameters seems to be less strong in SE than 2D-SWE, probably for the purely quantitative evaluation that is provided by 2D-SWE compared to SE. Moreover, despite the good intra and interoperator agreement [17,21,52], the variability that results from the external compression and that is not quantified is still a challenge [53]. 

Since the absence of flexural deformities was an inclusion criterion for selection and fore fetlock joints were evaluated in the standing horse with the leg in a weight bearing position to standardize the tissue state, we did not evaluate the degree of joint extension during the exam. Further studies should be carried out to assess the interaction between limb position, joint flexion, and 2D-SWE data.

The choice of the width and the positioning of the ROI is crucial for interpretation of the results. Since a small ROI was required in this case, a computerized analysis of pixel distribution was not necessary [17]. Moreover, the depth of the region to be examined allowed a correct placement of the region of interest, within values described in literature [51,54].

The criteria for the selection of the patients (clinical examination and radiographic/ultrasonographic results) and the absence of owner compliance for MRI to confirm OA represent the main of limitations the study. Moreover, in most cases, synovial fluid cytology or inflammatory markers analysis was lacking.

Future prospective and longitudinal studies should be planned for the evaluation of the 2D-SWE in severe cases of OA. As in tendon lesions, 2D-SWE of the distal attachment of the fetlock joint capsule data should be compared to MRI [22] and histology [55,56,57,58] and supported by cytologic or biochemical findings before a clinical validation of the technique could be established.

## 5. Conclusions

This was the first study to investigate 2D-SWE on equine joints in vivo. Here, 2D-SWE of the distal insertion of the joint capsule of the fore fetlock in horses is repeatable and reproducible, but, despite our positive expectations, we observed that is still not ready to be used in clinical settings for examination of the DJC. Differently from SE of the same region, it does not clearly differentiate joints affected by OA from healthy joints. Based on our findings, its poor sensitivity and specificity make the technique not suitable for application in clinical practice for the evaluation of DJC in fore fetlock joints. Despite these results, the promising data from previous studies [6,9,29,59,60,61] and their potential usefulness in the early detection of some disorders, assessment of disease progression or of the healing process as a response to treatment, make the technique worthy of further studies on the joints to establish a correlation with MRI findings or inflammatory markers in synovial fluid samples, to develop new applications or to improve those that are still under study.

## Figures and Tables

**Table 1:** Mean thickness of DJC in the Group H and P. Values are expressed as mean and standard deviation. Statistical significance was set at *p* < 0.05.

Variable	Group H	Group P
Left	1.05 ^a^	1.44 ^b^
Right	1.18 ^a^	1.47 ^b^

Different letters in the same row indicate significantly different results (*p* < 0.05).

**Table 2:** Velocity (m/s) and Young’s modulus (kPa) in Group S and Group P in longitudinal and transverse scans. Values are expressed as median and range. Statistical significance was set at *p* < 0.05.

Variable	Group H		Group P	
	Longitudinal	Transverse	Longitudinal	Transverse
m/s	6.53 (0.78–9.9) ^a^	8 (0.6–9.8) ^b^	7.01 (1.45–9.86) ^a^	7.43 (1.61–9.68) ^c^
kPa	125.86 (1.59–295.04) ^a^	191.56 (1.11–289.63) ^b^	146.36 (5.65–292.37) ^a^	164.64 (7.1–280.75) ^c^

Different letters in the same row indicate significantly different results (*p* < 0.05).

**Table 3:** Velocity (m/s) and Young’s modulus (kPa) in Group S and Group P in left and right limb. Values are expressed as median and range. Statistical significance was set at *p* < 0.05.

**Group H**	**Left Limb**		**Right Limb**	
	**Longitudinal**	**Transverse**	**Longitudinal**	**Transverse**
m/s	6.5 (0.78–9.9)	8 (1.93–9.67)	6.69 (0.96–9.83)	8.04 (0.6–9.8)
kPa	125.75 (1.59–295.04)	191.55 (10.66–281.28)	128.77 (3.38–289.51)	190.84 (1.11–289.63)
**Group P**	**Left Limb**		**Right Limb**	
	**Longitudinal**	**Transverse**	**Longitudinal**	**Transverse**
m/s	7.02 (2.4–9.86)	7.9 (1.91–9.43)	6.99 (1.45–9.25)	6.31 (1.61–9.68)
kPa	146.36 (15.5–292.37	187.59 (10.83–271.6)	143.84 (5.65256.03)	121.73 (7.1–280.75)

**Table 4:** Velocity (m/s) and Young’s modulus (kPa) in the unaffected limb and affected limb of Group P. Values are expressed as median and range. Statistical significance was set at *p* < 0.05.

Variable	Not Affected Limb of Group P		Affected Limb of Group P	
	Longitudinal	Transverse	Longitudinal	Transverse
m/s	6.94 (1.29–9.3) ^a^	7.65 (0.6–9.28) ^c^	7.09 (1.45–9.86) ^b^	7.43 (1.61–9.68) ^c^
kPa	143.72 (4.47–256.61) ^a^	175.34 (1.11–258.91) ^c^	149.8 (5.65–292.37) ^b^	164.64 (7.1–280.75) ^c^

Different letters in the same row indicate significantly different results (*p* < 0.05).

**Table 5:** Area under the curve (AUC) of the ROC curve for each variable. 95% CI (Confidence Interval) and optimal cut-off value are also shown.

	AUC		95%CI		Cut-off	
	Longitudinal	Transverse	Longitudinal	Transverse	Longitudinal	Transverse
m/s	0.517	0.616	0.4732–0.5609	0.571–0.661	6.61	7.55
kPa	0.518	0.61	0.4741–0.5618	0.565–0.655	130.75	184.3

## Data Availability

The data presented in this study are available on request from the corresponding author. The data are not publicly available due to privacy reasons.

## Supplementary Materials

The following supporting information can be downloaded at: https://www.mdpi.com/article/10.3390/vetsci9090478/s1, Table S1: Ultrasonographic score, modified from Yamada et al. [33].
