# Supplementary material for: 2D-SWE of the Metacarpophalangeal Joint Capsule in Horses
Source: Vet Sci. 2022 Sep 4;9(9):478. doi: 10.3390/vetsci9090478 (PMC9501397; doi:10.3390/vetsci9090478)
Supplement: Supplementary file 1 [file vetsci-09-00478-s001.zip › vetsci-1817023-supplementary.pdf]

Variables		Categories	Score
Subchondral surface appearance		Smooth	0
		Irregular	1
		Areas of depression	2
Synovial plica	Appearance	Normal	0
		Predominantly hyperechogenic	1
		Hyperechogenic calcification sites	2
	Size	Normal	0
		Increased up to 50%	1
		Increased by more than 50%	2
Joint capsule	Insertion	Smooth	0
		Discretely irregular	1
		Irregular	2
		Severe irregularity	3
	Appearance	Normal	0
		Localised hypoechogenic foci	1
		Hypoechogenic areas with hyperechogenic foci	2
	Thickness	Normal	0
		Increased by 20% in localised areas	1
		Increased	2
		Increased by more than 20%	3
Total (final sum)			14

Table S1: Table S1: Ultrasonographic score, modified from Yamada et al [33].
